# Growth and B-Phycoerythrin Production of Red Microalga *Porphyridium purpureum* (Porphyridiales, Rhodophyta) under Different Carbon Supply

**DOI:** 10.3390/microorganisms10112124

**Published:** 2022-10-27

**Authors:** Andrei B. Borovkov, Irina N. Gudvilovich, Irina A. Maltseva, Olga A. Rylkova, Yevhen I. Maltsev

**Affiliations:** 1 Department of Biotechnology and Phytoresources, A.O. Kovalevsky Institute of Biology of the Southern Seas of RAS, IBSS, 299011 Sevastopol; spirit2000sev@yandex.ru (A.B.B.); gudirina2008@yandex.ru (I.N.G.); ol.rylkova@yandex.ru (O.A.R.); 2 Faculty of Natural Sciences, A. Makarenko Melitopol State University, 72312 Melitopol; maltseva-irina22@yandex.ru; 3 Laboratory of Molecular Systematics of Aquatic Plants, K.A. Timiryazev Institute of Plant Physiology RAS, IPP RAS, 127276 Moscow, Russia

**Keywords:** *Porphyridium purpureum*, culture density, productivity, phycobiliproteins, B-phycoerythrin, protein

## Abstract

Red microalga *Porphyridium purpureum* (Bory) Drew is a well-known object of biotechnology due to its unique ability to synthesize a wide range of biologically active compounds. Enough minerals in an accessible form in a medium are a prerequisite for maintaining a high growth rate of *P. purpureum*. Carbon is the main element of microalgal biomass and is a component of all organic compounds. The work aimed to study the morphological features of cells and the accumulation and production of B-phycoerythrin and total protein in *P. purpureum* biomass in different ways of supplying CO_2_ into the culture. In Variant 1, CO_2_ was directly injected into a gas–air mixture (2–3 percent *v*/*v*) used for culture bubbling via capillary. In Variant 2, the air was supplied to the culture through the aquarium sparger. Variant 3 was like the first one but without the additional introduction of carbon dioxide. The application of the method for sparging atmospheric air led to a significant increase in both the productivity of the *P. purpureum* and the rate of protein and B-phycoerythrin synthesis in comparison with growing it using the air without spraying (two-and-a-half times, five times, and more than eight times, respectively). Moreover, there were significant changes in the morphological structure of *P. purpureum* cells, which were visualized both by microscopy and by changes in the color of the culture. Based on the experimental data obtained, the variants for the carbon supply experiment were ranked as follows: Variant 1 is better than Variant 2 and Variant 3. The use of atomization as a technological method made it possible to speed up the transfer of carbon dioxide from the air to the medium, which helped to keep the growth rate of *P. purpureum* biomass and B-phycoerythrin accumulation high.

## 1. Introduction

*Porphyridium purpureum* (Bory) Drew et Ross is the object of laboratory studies and mass cultivation [1,2,3,4,5]. The most commonly evaluated parameters that reflect the physiological state of the culture are population dynamics, photosynthetic pigment content, other biochemical characteristics, and morphological characteristics [6,7,8,9,10]. In practical terms, the resulting biomass of *P. purpureum* may be the source of several valuable physiologically active substances: extracellular sulfo-polysaccharides, unsaturated fatty acids (mainly eicosapentaenoic acid (EPA)), and pigments belonging to the phycobiliprotein (PBP) group [4,11,12,13,14,15]. For example, exopolysaccharides derived from *Porphyridium* have immunostimulating effects and are used in cosmetics and agriculture as growth stimulants. The most important medical application of EPA is prevention and correction of lipid metabolism disorders that lead to the development of atherosclerosis and several other cardiovascular diseases [3,11]. Due to its antioxidant and immune-stimulating activity, phycoerythrin pigment is widely used as a fluorescent dye and in health supplements [3,11,13].

As for the application aspect, the red pigment B-phycoerythrin (B-PE) number, which can reach over 12% of dry weight (85% of the total PBP), is of great interest [3,16]. The B-PE aqueous solution has a pink color and a strong orange fluorescence. The protein nature and lack of toxicity information generate excellent prospects for pigment usage in the food, cosmetics, and medical industries [4,13].

The relative content and production of B-PE in *P. purpureum* vary widely and depend on the influence of many factors, such as light conditions, nutrient bioavailability, salinity, temperature, and physiological state. However, light conditions and concentration of mineral nutrition elements are recognized as the main determinants of PBP photobiosynthesis processes [6,7,10,16,17,18]. The influence of nitrogen concentration on the rate of B-PE synthesis and its accumulation in *P. purpureum* cells is widely reported in the scientific resources [6,8,10,19]. B-PE content in *P. purpureum* cells is shown to be controlled by the concentration of nitrogen in the culture medium. After this element of mineral nutrition is exhausted, rapid pigment degradation is observed [1,2,6,8,19]. Carbon is also one of the most critical mineral elements; it is a constituent of all organic compounds and is the main element of microalgal biomass [12,20,21,22,23]. Atmospheric CO_2_ is the primary carbon source for plankton microalgae as most autotrophic microorganisms use inorganic carbon during photosynthesis [22,23,24]. At low CO_2_ concentrations, *P. purpureum* photosynthetic efficiency improvement is observed due to a CO_2_ concentrating mechanism [3,23]. However, a sufficient amount of carbon in an accessible form in the medium is a prerequisite for high-speed photobiosynthetic processes in *P. purpureum* cells and the intensification of its biomass yield [15].

Scientists discuss two main ways of enriching nutrient media with inorganic carbon: direct bubbling CO_2_ into the medium and additional application of sodium hydrocarbonate NaHCO_3_ [2,5,15,16,21,22]. During the intensive cultivation of *P. purpureum*, the supply of carbon is usually achieved by adding CO_2_ to the air mixture (up to 3–5% by volume). However, the prospect of carbon dioxide being used in large-scale cultivation of *P. purpureum* inevitably leads to an increase in the cost of the produced biomass. As an alternative, one should consider the increase in the efficiency of using CO_2_ in the ambient air. To intensify the process of CO_2_ dissolving from the air in the aquatic medium, it is necessary to create favorable conditions for this: to increase the contact surface of the air–liquid medium phases (for example, in the case of air atomization) and to maintain a pH of 8.0–8.3 units, optimal for preferential placement of bicarbonate ion in the media, preferably absorbed by photosynthetic microorganisms [22,23].

Thus, selecting a simple and inexpensive method for introducing a carbon source into a culture of microalgae to maintain the optimum quantity of it in a culture medium without mineral and light limitation is decisive for intensive microalgae cultivation and synthesis of protein substances. The information about morphological features of the cells helps to identify patterns of culture development under different growing conditions. Therefore, the goal of the work was to study the morphological characteristics of the cells, the accumulation and production of B-PE, and the total protein in the culture of *P. purpureum* in various ways of introducing CO_2_ into the culture.

## 2. Materials and Methods

The research was carried out on the base of the Department of Biotechnology and Phytoresources (FRC IBSS, Sevastopol). The object of the study was the culture of red microalga *Porphyridium purpureum* (Rhodophyta) strain IBSS-70 [18] from the Collection of Hydrobionts of the World Ocean from the Scientific and Educational Center for Collective Use (FRC IBSS). Cultivation was carried out on a nutrient medium for marine red algae according to Trenkenshu [25] with the following composition: NaNO_3_ 1.2 g/L, NaH_2_PO_4_ × 2H_2_O 0.45 g/L, EDTA-Na_2_ 0.037 g/L, FeC_6_H_5_O_7_ × 3H_2_O 0.0265 g/L, MnCl_2_ × 4H_2_O 0.004 g/L, Co(NO_3_)_2_ × 6H_2_O 0.0031 g/L, (NH_4_)_6_Mo_7_O_24_ × 4H_2_O 0.009 g/L, K_2_Cr_2_(SO_4_)_2_ × 4H_2_O 0.0017 g/L. The medium was prepared in sterilized seawater (Black Sea water with a salinity of about 18‰). *P. purpureum* culture was grown in 5 × 25 × 50 cm flat-plate glass photobioreactors with an active layer of 5 cm and volume of 3 L, and the inoculum/nutrient medium ratio was 1:4.2 *v*/*v*. The light source used was a light grid of 18-watt fluorescent lamps; the average light on the surface of photobioreactors was 10,000 lx. The light intensity on the surface of the photobioreactor was recorded with the Yu-116 luxmeter with an error of not more than 5% of the measured value. The temperature was maintained at 26–28 °C and 8–9 pH. Culture bubbling was carried out by air with the aquarium compressor Hailea ACO-308. The air supply speed was the same for all photobioreactors and was about 0.8 L/L of culture per minute. In Variant 1, the bubbling was carried out through a glass tube with an internal diameter of 4 mm and an additional introduction of CO_2_ (2–3% *v*/*v*); in Variant 2 through an aquarium sparger device in the form of a plastic tube with a length of 5 cm and a diameter of 5 mm, in which the diameter of the pores did not exceed 0.1 mm; in Variant 3 (control) similar to Variant 1 but without the additional introduction of carbon dioxide.

For microscopic studies, the culture of *P. purpureum* was fixed by glutaric aldehyde to a final concentration of 2.5% in a sample for 1 h. An aliquot of a fixed suspension of microalgae (0.05 mL) was placed on the object glass, covered with a cover, and examined in the Central Control Station “Molecular Structure of Matter” of SevGU with the help of a microscope Mikmed-6 (“LOMO”, Russia) with a digital camera (MS-6.3, “LOMO”, Russia) and computer software (MCVeiw, “LOMO-Microsystems”, Russia) at an increase of ×1000. Cell sizes were determined by microphotography using Image J 1.50i (National Institutes of Health, Bethesda, MD, USA, Java 1.6.0_20 (32-bit). At least 50 cells were measured in 10–20 fields of view. When preparing samples for scanning electron microscope (EMS), an aliquot of the fixed sample (1–2 mL) was concentrated on a track membrane with a pore diameter of 2.0 μm (produced by JINR, Dubna, Russia). Then, dehydration was carried out using a series of ethanol: 20%, 30%, 50%, 75%, 96%, and 100% [26]. The Leica EM CPD300 (Wetzlar, Germany) was used to dry the samples at a critical point (1.5–2.5 h). For spraying (Au/Pd; 0.5–1.0 min.), the device Leica EM ACE200 (Germany) was used. The samples obtained were viewed with the help of SEM Hitachi SU3500 (Tokyo, Japan) when zooming by ×7000.

The dry weight (DW) content in the culture was determined by photometric and also by weight methods [27]. Microalgal suspension optical density at 750 nm (D_750_) was measured by Unico 2100 photometer (United Products & Instruments, Dayton, NJ, USA) in 5 mm pathway cuvettes; absolute measurement error did not exceed 1.0%. Optical density units (o.d.u.) (D_750_) conversion to dry biomass weight (DW) values were expressed as follows:

DW = k × D_750_,

where DW is dry biomass weight, D_750_ is culture optical density, k is the conversion factor. Empirical conversion factor (k = 1.4 g/L × o.d.u.^−1^) was defined previously.

Aliquots of 10 mL algal suspension were filtered through precombusted (105 °C, 24 h) nitrocellulose filters (Sartorius, 25 mm, nominal pore size 5 μm). Filtered algal samples were washed with 20 mL of distilled water. The filters were dried at 105 °C to a constant weight, cooled down in a vacuum desiccator, and then weighed [28].

After careful mixing, the PBP and protein samples were taken at different growth phases of the batch culture (at least every two days). The *P. purpureum* culture suspension obtained in the experiment was centrifuged at 4000 rpm for 10 min, the pressurized liquid was drained, and the deposited biomass was frozen and used to determine the PBP by the spectrophotometric method [29]. To quantify B-PE, *P. purpureum* biomass was added to phosphate buffer (0.05 M; 7–7.5 pH) and placed alternately at −20 °C and 4 °C for 24 h for repeated freeze-thawing. The spectra of the pigment extracts were measured at the SF-2000 registering spectrophotometer (ZAO “OKB SPECTR”, Saint-Petersburg, Russia) in a wavelength range of 400–800 nm with a pitch of 0.1 nm. The optical density of the extracts produced was recorded in the field of characteristic absorption maxima B-PE (545 nm), R-phycocyanin (R-PC) (615 nm), and allophycocyanin (APC) (650 nm), and at 750 nm (to take into account non-specific solution absorption). The concentration of pigments in the aqueous extract was calculated by Ref. [29] using optical density values for the corresponding wavelengths.

Protein content was determined by Lowry [30]. Protein extraction from *P. purpureum* was performed using 2 mL of culture, centrifuged (4000 rpm, 15 min), and washed with distilled water. The obtained pellet was resuspended in 2 mL of NaOH 1 M for 1 h at 100 °C. The extract was centrifuged at 4000 rpm, 15 min, and 200 µL of the supernatant was taken to carry out the protein analysis described in Ref. [30].

Triplicate biological and analytical samples were used in the experiments. Arithmetic means ( $\bar{x}$ ), standard deviations (SD), error of the mean, and confidence intervals for the mean (Δ $\bar{x}$ ) were calculated. All calculations were carried out in Libre Office and Scidavis software for the level of importance of α = 0.05. The tables and graphs show the three repetitions’ average values and calculated confidence intervals ( $\bar{x}$ ± Δ $\bar{x}$ ).

## 3. Results

The ultimate productivity of algal culture using atmospheric CO_2_ as a carbon source was previously assessed [31]. To verify the obtained values, experimental studies were conducted to grow the microalgae *P. purpureum* on the sprayed atmospheric air. In comparison, culturing regimes with air bubbling and the additional introduction of CO_2_ into the air/gas mixture were selected, which fully meet the carbon requirements of the microalgae culture and maximize its production values.

**Figure 1:**
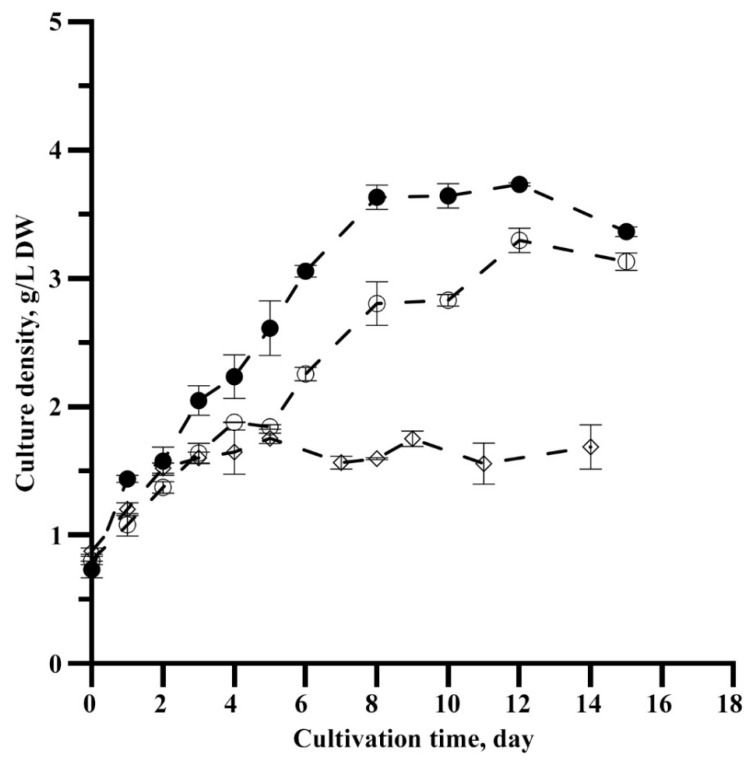
The density of *P. purpureum* culture under the different methods of carbon introduction into photobioreactor: ●—additional introduction CO_2_; ○—air spraying; ◊—air bubbling through a capillary (the data are means of three replicates ± Δ $\bar{x}$ ).

During the growing period, the density of the *P. purpureum* culture increased by five, four, and one-and-a-half times (for Variants 1, 2, and 3, respectively) (Figure 1).

The *Porphyridium* biomass could have been increased by about 3 g/L over the growing period considering the nitrogen concentration in the medium and dilution [1]. Similar values to the calculated ones of this parameter were observed for the CO_2_ injection and sparger experiment variants. For Variant 3, the biomass growth was more than four times lower than estimated (Table 1).

**Figure 2:**
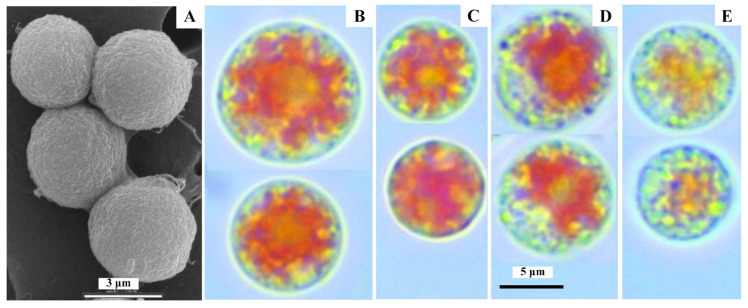
Cells of *P. purpureum* culture under the different methods of carbon introduction into photobioreactor: (**A**)—the initial stage of cultivation according to SEM; (**B**)—the same according to light microscopy; (**C**)—additional introduction CO_2_ (6th day); (**D**)—air spraying (6th day); (**E**)—air bubbling through a capillary (6th day).

The *P. purpureum* cells had a circular shape, no deformation, and a diameter ranging from 5.9 to 11.5 μm (7.9 ± 0.8 μm on average) at all stages of culturing (Figure 2A–E).

At the beginning of the experiment, *P. purpureum* cells had a bright red coloring due to B-PE in chloroplasts, which uniformly occupied virtually all the intracellular space (Figure 2B). The cell size ranged from 7.6 to 11.5 μm (the average cell size was 9.0 ± 1.3 μm). After 6 days of growing *P. purpureum* with the addition of CO_2_, the appearance of the microalgae cells did not differ much from the initial; visible color changes were not observed. However, smaller cells were more common, resulting in a 15% decrease in average cell size (to 7.6 ± 0.4 μm) compared to the original (Figure 2C). By the sixth day, when *P. purpureum* was grown in the air spraying, its cells also had reddish coloration, which did not extend to all the intracellular space. For example, about a third of the cell content was olive-yellow. The observed morphological changes appear to have been caused by the onset of degradation of the pigment complex (Figure 2D).

Besides, there was a slight retraction of cytoplasm from the membrane’s inner surface and a tendency to increase cell size compared to the starting cell; their average size was 9.6 ± 0.2 μm. The most significant changes in the morphological structure of *P. purpureum* cells have been observed during its growth using air without air spraying: a substantial proportion of the cells in the culture were small (the average cell size decreased by 13% from the original to 7.8 ± 0.8 μm), chloroplasts in most cells were olive-yellow in color, and cytoplasm retraction was pronounced. In addition, significant heterogeneity in cell size structure with diameters ranging from 5.9 to 8.8 μm (the presence of both small and large cells in *P. purpureum* culture) was observed in this experimental variant compared to the other two variants.

**Figure 3:**
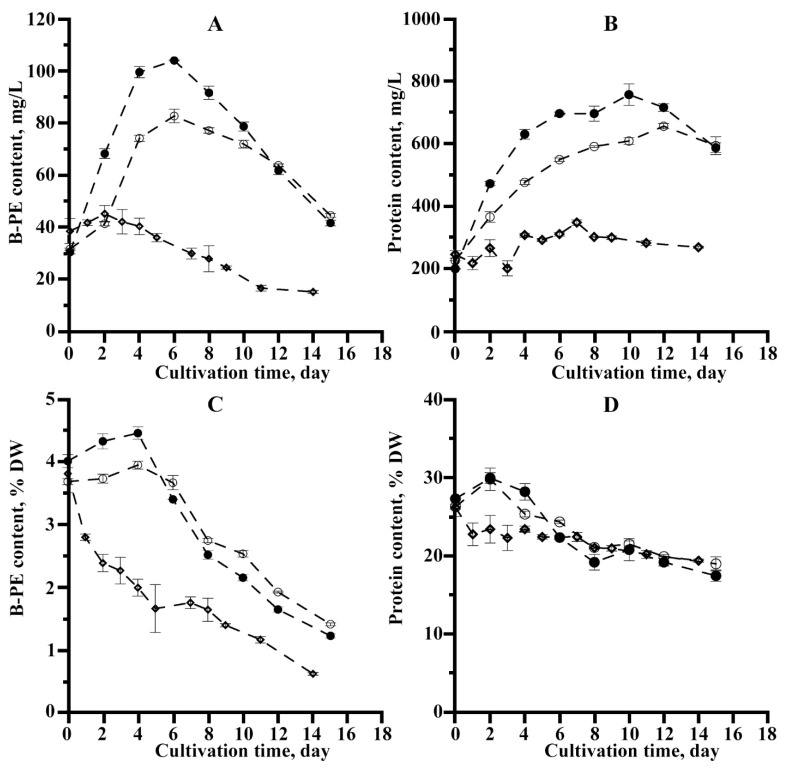
B-PE (**A**) and protein (**B**) content in *P. purpureum* culture and its biomass ((**C**,**D**), respectively) under the different methods of carbon introduction into photobioreactor: ●—additional introduction CO_2_; ○—air spraying; ◊—air bubbling through a capillary (the data are means of three replicates ± Δ $\bar{x}$ ).

In addition to the change in the culture density during *P. purpureum* batch cultivation, there was also a change in B-PE and protein content in both cultures and their cells (Figure 3).

The contents of both protein and B-PE in the cells and culture of *P. purpureum* at the time of the experiment were approximately the same for all three variants (Figure 3A,B). The B-PE content change for Variants 1 and 2 in both culture and *P. purpureum* cells was similar: initially, there was an increase in its content, and, after 6 days, there was a decrease. Thus, the content of B-PE in the culture increased 3.4 and 2.6 times in 6 days for Variants 1 and 2, respectively. Increasing the B-PE content in cells was less pronounced: for example, in 4 days, it was 11 and 8% for Variants 1 and 2, respectively. For Variant 3, a significant decrease in B-PE content in both biomass and *P. purpureum* culture (2 and 1.3 times in 6 days, respectively) was observed (Figure 3A,B). It should be noted that the B-PE content in *P. purpureum* cells in Variants 1 and 2 by the sixth day of cultivation was 2-fold higher than in the control (Figure 3C), confirming the above-noted visual differences in cell culture coloration (Figure 2C–E).

The protein content of *P. purpureum* culture for Variants 1 and 2 by 10 days increased by 3.8 and 2.7 times, respectively, amid a similar increase in culture density. For Variant 3, there was a 1.4-fold increase in protein in *P. purpureum* culture over 7 days of growing, which also primarily correlated with the change in culture density in this experiment variant (Figure 3B). The protein content in the microalgae biomass declined by 18–20% by the 10th day for all experiment variants (Figure 3D). It should be noted that the nature of the protein change in *P. purpureum* culture for all the variants was mainly determined by the culture density dynamics. The reduction in its content in microalgae cells was less significant than in B-PE.

Based on the obtained data, the average rate of B-PE and protein synthesis in the culture of *P. purpureum* was calculated (Table 2).

The maximum observed B-PE synthesis rate of *P. purpureum* culture was recorded for a variant of the experiment with direct CO_2_ addition. When air spraying was conducted in the culture, the synthesis rate of this pigment was lower by 30%, and, in the absence of spraying and air bubbling, the B-PE synthesis rate was significantly reduced to negative values (the B-PE concentration decreased more than twice from its initial value by the sixth day; therefore, the observed production had a negative value; i.e., destruction processes prevailed over synthesis processes). A similar pattern was observed for the standard protein synthesis rate.

**Figure 4:**
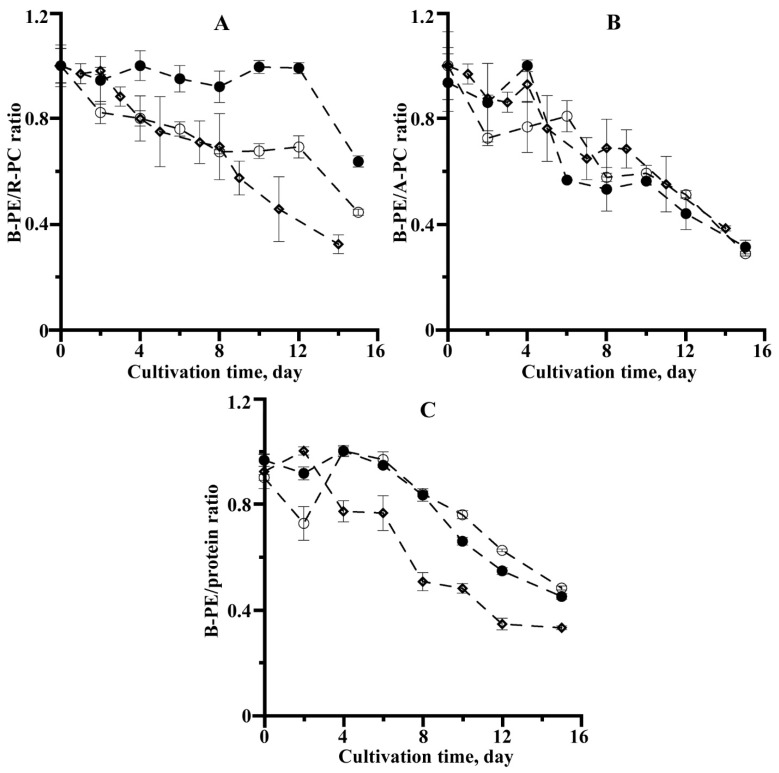
B-phycoerythrin/R-phycocyanin (**A**), B-phycoerythrin/Allophycocyanin (**B**), and B-phycoerythrin/protein ratio (**C**) normalized by maximum values of *P. purpureum* culture under the different methods of carbon introduction into photobioreactor: ●—additional introduction CO_2_; ○—air spraying; ◊—air bubbling through a capillary (the data are means of three replicates ± Δ $\bar{x}$ ).

Throughout cultivation, quantitative changes in PBP were observed, and the composition changed (Figure 4).

In the experiment conducted, mainly the numerical values of the ratios of the PBP itself (on average, 2.5–3 times) and the balance of B-PE/protein (2–2.7 times) in the process of cultivation (Figure 4) were decreased, with the maximum reduction in the ratios of B-PE/R-PC and B-PE/protein being observed in the variant of the experiment with air bubbling. It should be noted that the B-PE/R-PC ratio of *P. purpureum* is stable and remained at the original level for 12 days of cultivation with additional CO_2_ injection into the medium (Figure 4A). The B-PE/APC ratio change was broadly similar in all the experimental variants.

## 4. Discussion

As the microalgae cultures grew and the cell numbers increased, their illuminance decreased due to self-shading. However, the maximum rise in *P. purpureum* biomass in the experiment was noted in Variant 1, where the light limiting of culture growth was most pronounced. In this case, the rate of *Porphyridium* growth in the investigation could be determined not by the level of illumination of the culture cells but by the number of biogenic elements. As the starting concentration of mineral nutrition elements was the same in all the experimental variants and the conditions for culturing differed only in how carbon was introduced into the culture, the growth rate of *P. purpureum* appeared to have been determined by the amount of carbon in the medium. CO_2_ was additionally supplied in Variant 1, which predictably provided more favorable culture growth conditions. In Variant 2, the sparger application significantly reduced the size of the air bubbles filled to the cultivator, which increased the liquid–gas surface area. This technological method was presumably supposed to increase the amount of CO_2_ transferred to the culture medium. The conditions for carbon source transition into the liquid phase in Variant 3 (control), in the absence of direct CO_2_ addition and sparging devices, were the most unfavorable.

It was shown previously that, if microalgal cultures had a higher growth rate, their cells were smaller [32]. Our results also support this: the growth rate of *P. purpureum* was 30% lower when using sparger than CO_2_, while the average cell size of the sparger variant was 26% higher. Caused by insufficient carbon supply, culture growth rate reduction resulted in a reduced cell division rate and increased cell size. In contrast to the other two variants, Variant 3 showed pronounced heterogeneity in cell size structure (the presence of both small and large cells in the *P. purpureum* culture). This may indicate significantly unfavorable conditions under which the energy derived from light reactions was mainly spent on maintaining cell homeostasis rather than on the cell division process [33]. Furthermore, it should be noted that, in Variants 1 and 2, the *P. purpureum* culture was still in the active growth stage on the sixth day of cultivation. In contrast, the growth rate in Variant 3 (control) was significantly lower (Figure 1, Table 1). The culture was already in the stationary growth phase, which did not allow a sufficiently correct comparison of the obtained size data on the sixth day.

The concentrations of mineral nutrition elements and light conditions significantly affect the PBP content in the cells and microalgae cultures. Under negative influences (e.g., increased illumination, mineral nutrient deficiencies), the culture growth rate inevitably changes, and the photosynthetic apparatus of microalgae cells undergoes a significant transformation: the content of PBPs, such as B-PE, and R-PC, is reduced. Furthermore, their ratios are altered [8,10,16,34,35]. The greatest changes are observed for PE, a protein-based light-harvesting pigment [6,17,34].

The decrease in B-PE in *P. purpureum* cells during batch cultivation is noted by many researchers. It is explained by the direct dependence of its quantity on the concentration of nitrogen in the medium [2,6,8,19]. For example, at low (3.5 mM KNO_3_) and medium (5.9 mM KNO_3_) nitrogen concentrations in the medium, the maximum B-PE content was recorded at the start of the experiment (6.41% DW) and reduced over 16 days to 0.22% DW and 0.48% DW, respectively [8]. At high concentrations of nitrogen in the medium (17.6 mM KNO_3_), the highest B-PE content was observed on the sixth day (8.18% DW) [8]. However, on the 16th day, the concentration of B-PE also decreased significantly (to 2.36% DW) [8]. Thus, the B-PE content in the early stage of cultivation was considerably higher than in the middle and late stages due to the depletion of nutrients (primarily nitrogen) during *P. purpureum* batch cultivation. The pigment destruction level was directly related to the initial nitrogen concentration in the medium. At a low nitrogen concentration, the PE content declined to shallow values by the end of the cultivation [8,34]. Although the initial nitrogen concentration was similar in all three experimental variants, the pattern of B-PE content change in *P. purpureum* cells (Figure 3C) generally correlated with the data provided above on B-PE content change at the different nitrogen concentrations [8]. As the nitrogen concentration did not vary in our experiment, the observed quantitative changes in B-PE content during cultivation (Figure 3C) can be explained by different amounts of carbon in the medium according to different ways of its introduction into *P. purpureum* culture.

Besides nitrogen, carbon is also an indispensable element of mineral nutrition for microalgae growth and metabolism [5,16,20]. The results indicate that the amount of carbon entering the nutrient medium had a significant effect on the photosynthetic apparatus of *P. purpureum* cells, which is consistent with references on the considerable impact of carbon concentration and the different C/N ratio in the medium on B-PE concentration in *P. purpureum* [5,16]. The observed morphological changes in *P. purpureum* cells support earlier studies regarding the effects of carbon dioxide on the pigment and membrane composition of cyanobacteria. Thus, after the CO_2_ supply to *Thermostichus lividus* Copeland) Komárek et Strunecky culture was stopped, chlorophyll *a* and C-phycocyanin in its cells were destroyed, and the culture was consequently yellowed [20]. It has been shown that this process is reversible: the resumption of CO_2_ production led to rapid recovery in synthesizing these pigments and the return of the original culture color [20]. The addition of sodium bicarbonate as a source of carbon in the ASW nutrient medium also affected the content of significant phycobiliproteins in *P. purpureum*. Thus, when 2 g/L NaHCO_3_ was added, the B-PE, R-PC, and APC contents in its cells increased by 20, 45, and 35%, respectively [16]. Lower light intensity and higher concentrations of NaHCO_3_ in the culture contributed to higher levels of phycobiliprotein accumulation in *P. purpureum*: the maximum B-PE content was 12.17% [16].

The total protein in red microalgae *P. purpureum* may be 28–41% in the dry algae mass. Its content is predominantly affected by light intensity, nitrogen concentration, and culturing duration [12,34]. The specifics of *P. purpureum* protein change in the experiment generally coincided with the direction of B-PE, which corresponds to the current understanding of the correlation between the amount of total protein and the pigments that make up the protein complexes [35]. It should be noted that the restriction or lack of carbon in the medium of all organic compounds usually leads to a limitation in *P. purpureum* biomass concentration, a shift in biosynthesis direction, and the accumulation of a particular product [5].

Many factors may influence PBP ratios, the most important of which are light conditions and mineral nutrition [12,16,36]. For example, low nitrogen concentrations in the medium reduced the ratio of B-PE to R-PC faster than in medium and high concentrations. At the end of cultivation, the B-PE/R-PC ratio was 2.25, 2.95, and 5.71 for groups with low, medium, and high nitrogen concentrations, respectively, with a starting value of 6.75 for all the variants [8]. The addition of carbon to the nutrient medium has been known to significantly alter the content of significant phycobiliproteins in *P. purpureum* and their ratios [16]. It should be noted that the sharp decrease in the B-PE/R-PC and B-PE/APC ratios indicates that B-PE is more sensitive to changing medium conditions than R-PC and the APC is most stable [8,17]. This is also consistent with the experimental data: the stability of the B-PE/R-PC ratio if CO_2_ is introduced into the culture appears to be due to this variant’s better carbon conditions than in other experiments (Figure 4A). In addition, the ratio of PE/APC (or PC/APC) in microalgae and cyanobacteria controls the transfer of energy from phycobilisomes to chlorophyll *a* [35]. The results of the phycobilisomes degradation are a reduction in the size of the complexes, which begins at the periphery, and a reduction in the ratio of light-harvesting pigment to the APC at the center of the antenna structures [14,37].

*P. purpureum* has previously been shown to be as productive as 0.5 g/L × day and 40 mg/L × day by biomass and B-PE, respectively [6,19]. The values obtained in the experiment do not reach this level of productivity in biomass and B-PE (0.36 g/L × day and 12.3 mg/L × day, respectively), which can be explained by fundamental differences in the approaches to the organization of accumulation and semi-continuous cultivation methods. However, the B-PE productivity of *P. purpureum* obtained in the CO_2_ addition experiment (Variant 1) is comparable to the productivity values obtained in the semi-continuous mode under similar conditions (13 and 15 mg/L × day, respectively) [6].

Regarding the productivity of the *P. purpureum* culture grown with air spraying, previous studies have shown that the average productivity of the *P. purpureum* culture in the linear growth area was 0.29–0.37 g/L × day and 10.3–17.5 mg/L × day of B-PE, which is comparable to the data obtained in the experiment (Variant 2) [8,18]. Moreover, similar data were received in the cultivation of the *Dunaliella salina* culture using air spraying: the average productivity was 0.24 g/L × day [31].

The absence of additional CO_2_ in the gas–air mixture and the use of sparger elements during the cultivation of *P. purpureum* resulted in a reduction in the rate of culture growth by 2.5–3.5 times, as well as a reduction in the rate of synthesis of B-PE and protein in cells by more than five times. In addition, changes in the morphological structure of its cells were observed, which were visualized both by microscopy and culture color changes, which also confirm the predominance of B-PE degradation processes in *P. purpureum* cells under these conditions. Based on the obtained experimental data on the productivity of *P. purpureum* culture by biomass, B-PE, and protein, the variants of the experiment on the sufficient amount of carbon in the medium in an accessible form were arranged in the following way: Variant 1 was better than Variant 2 and Variant 3 (Table 1 and Table 2). The results showed that introducing CO_2_ into the culture medium significantly affected the concentration of B-PE pigment in *P. purpureum* and often determined its growth and production characteristics. The application of atmospheric air spraying technology led to an improvement in the carbon supply of the *P. purpureum* culture and an increase in its average productivity in biomass, B-PE, and protein by 2.5, 8.5, and 6.5 times, respectively, compared to its productivity in non-atomized cultivation (Table 1 and Table 2).

## 5. Conclusions

It was experimentally shown that the carbon content of *P. purpureum* culture is one of the most important factors determining its production characteristics. Although the starting amount of mineral nutrition elements in the *P. purpureum* culture was sufficient to ensure high rates of culture growth and B-PE synthesis in all the experimental variants, the carbon supply conditions significantly limited these parameters. The absence of additional CO_2_ in the gas–air mixture and the use of sparger elements in the cultivation of *P. purpureum* led to a sharp decrease in the rate of culture growth and the rate of B-PE synthesis and, in general, photobiosynthetic processes in the cells, which were visualized both by morphological changes in the cells and by changes in the color of the culture. The use of the technological method for atomization made it possible to intensify the processes of dissolving atmospheric air carbon dioxide in the medium and to significantly increase the productivity of the *P. purpureum* culture and the rate of B-PE synthesis without the additional use of CO_2_. Red microalga *P. purpureum* is a typical single-cell photosynthetic autotrophic microorganism, and the availability of carbon sources in an accessible form is undoubtedly a critical factor in its growth and metabolism. The results demonstrate the possibility of significantly reducing or eliminating the CO_2_ supply during *P. purpureum* cultivation, whereas varying the carbon supply method makes it possible to manage the cultivation system performance and the accumulation of biologically valuable substances.

## Figures and Tables

**Table 1:** The productivity characteristics of *P. purpureum* batch culture under the different methods of carbon introduction into photobioreactor (the values are means of three replicates ± Δ $\bar{x}$ ).

Variants	Productivity			Yield, g/L
	Ultimate, g/L × Day [31]	Maximum, g/L × Day	Average (for 8 Days), g/L × Day	
CO_2_ introduction	–	0.41	0.36 ± 0.017	3.00 ± 0.05
Air spraying	0.37	0.27	0.25 ± 0.015	2.50 ± 0.06
Air bubbling		0.24	0.10 ± 0.011	0.68 ± 0.04

**Table 2:** The productivity characteristics of *P. purpureum* batch culture on B-PE and protein under the different methods of carbon introduction into photobioreactor (the values are means of three replicates ± Δ $\bar{x}$ ).

Variants	Productivity	
	Average of B-PE (for 6 Days), mg/L × Day	Average of Protein (for 6 Days), mg/L × Day
CO_2_ introduction	12.3 ± 0.8	82.8 ± 4.1
Air spraying	8.5 ± 0.7	53.9 ± 0.8
Air bubbling	−0.5 ± 0.01	10.8 ± 0.5

## Data Availability

The data presented in this study are available on request from the corresponding author.
